# Neuroimaging changes in major depression with brief computer-assisted cognitive behavioral therapy compared to waitlist

**DOI:** 10.1038/s41380-025-02945-x

**Published:** 2025-03-11

**Authors:** Yvette I. Sheline, Michael E. Thase, Elizabeth A. Hembree, Nicholas L. Balderston, Frederick J. Nitchie, Alexandra S. Batzdorf, Walid Makhoul, Kevin G. Lynch

**Affiliations:** 1 Center for Neuromodulation in Depression and Stress, University of Pennsylvania, Philadelphia, PA USA; 2 Department of Psychiatry, University of Pennsylvania, Philadelphia, PA USA

**Keywords:** Depression, Diseases

## Abstract

The goals of the current study were to determine the efficacy in major depressive disorder (MDD) of a shortened, computer-augmented cognitive behavioral therapy (CCBT) protocol and to determine brain plasticity effects following CCBT. Seventy-two MDD participants were randomized to CCBT or waitlist control groups and compared to 40 healthy controls (HCs). Functional MRI data were collected for all participants and repeated for patients following CCBT (five therapist-administered manualized CBT sessions plus computer training exercises). Linear mixed-effects models evaluated changes in depression scores throughout treatment and in connectivity from pre- to post-CCBT. Linear regression models compared connectivity differences between groups (MDD vs. HC). Following CCBT, there were decreases in MADRS and BDI (*p*s < 0.001); there was more negative connectivity of dlPFC with sgACC and DMN with sgACC (*p*s < 0.002); and there was more positive connectivity of FPN with nucleus accumbens, bilateral amygdalae, bilateral hippocampi, and sgACC and of DMN with ventral and dorsal bilateral anterior insulae (*p*s < 0.01). There were no associations between change in MADRS and change in connectivity; however, there was an association between change in BDI and change in FPN–sgACC connectivity (*p* = 0.01). A shortened CBT schedule coupled with home computer exercises was associated with decreased depression symptoms and augmented PFC connectivity with multiple subcortical regions. One possible mechanism of the CCBT intervention is modulating PFC connectivity with subcortical regions, influencing top-down control of affective processes dysregulated in MDD.

## Introduction

Cognitive behavioral therapy (CBT) is the psychotherapy approach with the best evidence base for the treatment of depression [1, 2]. It has established efficacy for major depressive disorder (MDD) in clinical trials in comparison to both attention-control conditions and antidepressant pharmacotherapy [1, 2]. CBT is equally as efficacious as antidepressant medication therapy [3, 4] but with more enduring effects [4, 5].

A key objective of CBT is to train patients with depression to improve voluntary mood regulation using techniques that include cognitive restructuring, behavioral activation, and reappraisal, as detailed in “Cognitive Therapy of Depression,” [6] which has been used in most recent outcome studies of CBT for depression [7–9]. An extension of this training has been the development of web-based CBT interventions [10, 11]. The utility of computer-augmented models of therapy (CCBT) such as *Beating the Blues* and *Mood Gym* has now been established by more than two dozen randomized controlled trials [10, 12, 13]. The *Good Days Ahead* (GDA) [14] model of CCBT used in this study was developed primarily to serve as an adjunct to therapy, with the goal of complementing and streamlining the efforts of therapists.

The CBT-induced brain mechanisms for therapeutic efficacy have been described in previous studies examining treatment-induced changes in both brain activation and functional connectivity. In a previous study examining the effects of CBT [15, 16], we delivered manualized individual weekly CBT psychotherapy sessions over a 12-week period by an expert clinical psychologist. That study found that the majority of patients responded to treatment with a greater than 50% reduction in mean Montgomery-Åsberg Depression Rating Scale (MADRS) [17]. In addition, the reduction in MADRS was correlated with increased amygdala connectivity with prefrontal cortex (PFC) using longitudinal functional principal components analysis. We also used traditional whole-brain, voxel-wise regression among patients and found that, after adjusting for age, sex, and motion, within the frontoparietal network (FPN), there was an increase in functional connectivity between the amygdala and inferior frontal gyrus (IFG). The same study also showed increased activity in the dorsolateral prefrontal cortex (dlPFC) associated with depression improvement following CBT [16].

The goal of the current study was three-fold: first, to determine in a prospective study whether, as opposed to a waitlist control period, the web-based program GDA combined with only five therapist-guided CBT sessions would be associated with improved depression symptoms. Second, we aimed to examine changes in functional connectivity during GDA and waitlist, and specifically to determine whether cortical–subcortical connections would change and what pathways would be involved. Third, we examined associations between change in connectivity and change in depression symptomatology and whether this differed between treatment responders and non-responders.

## Methods

### Participants

Participants (*N* = 112) were recruited from within the University of Pennsylvania and the surrounding community. Of those, 78 (70%) were female and 34 (30%) were male. Participants with MDD were primarily recruited from the outpatient clinics of the University of Pennsylvania, including the Mood and Anxiety Disorders Program, Student Health, the general adult psychiatry programs, and other Penn-affiliated clinics as well as from the surrounding community in Philadelphia. Our target sample size was a net, following exclusion/attrition, of 40 healthy controls (HCs) and 60 people with MDD. Sample size was selected to have sufficient power (>80%) to detect changes in connectivity following CCBT based on pilot data, including mean ± SD changes of 0.09 ± 0.05 between FPN and limbic regions and 0.08 ± 0.04 between dlPFC and subgenual anterior cingulate cortex (sgACC).

MDD inclusion criteria were: (1) Ages 18–60 years, gender-inclusive; (2) DSM-5 diagnosis of MDD in a current major depressive episode (MDE) as assessed using the Structured Clinical Interview for DSM-5 (SCID-5) [18]; (3) MADRS score ≥ 20; (4) Capacity to provide informed consent; and (5) Regular access to a home computer, tablet, or phone.

MDD exclusion criteria were: (1) Unstable medical conditions; (2) Alcohol or substance use disorder within the past six months; (3) Diagnosis of the following concurrent DSM-5 (SCID-5) psychiatric disorders: any psychotic or organic mental disorder, bipolar disorder, primary anxiety disorder, or primary eating disorders; (4) Pregnancy; (5) A neurological disorder or prior head trauma with evidence of cognitive impairment; (6) Contraindication to CBT as primary treatment for depression, including prior non-response to an adequate trial of CBT (at least eight weeks with credentialed CBT therapist), or suicidal ideation including a plan; (7) Currently receiving psychotropic medication; or (8) Unable to tolerate or contraindication to MRI.

HC inclusion criteria were: (1) Ages 18–60 years; (2) Lack of current or past DSM-5 mental health or substance use diagnosis, as assessed by the SCID-5 and a MADRS score of < 8; and (3) Capacity to provide informed consent. Healthy control exclusion criteria included items 1, 2, 4, 5, 7, and 8 from the MDD exclusion criteria.

#### Ethical approval and consent to participate

All participants signed an informed consent form and the protocol was approved by the Institutional Review Board for human subject research at the University of Pennsylvania (IRB Protocol Number 832295). All procedures contributing to this work comply with the ethical standards of the relevant national and institutional committees on human experimentation and with the Helsinki Declaration of 1975, as revised in 2007 [19].

### Procedure

Once enrolled in the study, participants who met criteria for an MDE were randomized by random number generator to either the early group (initiating treatment within the first week) or the waitlist group (initiating treatment after a three- to four-week waitlist period). All participants were told that scheduling the start of CCBT would take one to four weeks. Clinical assessments and MRI were performed at baseline. For the waitlist group, a second baseline was performed after approximately three to four weeks of no study activity, constituting the waitlist period. After the waitlist period, clinical assessments and MRI were repeated. MADRS, Beck Depression Inventory–II (BDI) [20], 17-item Hamilton Depression Rating Scale (HDRS) [21], and Insomnia Sleep Index (ISI) [22] scores were used as measures of pre- and post-treatment depression severity. MADRS was collected at each study visit; BDI and ISI were collected at baseline, treatment initiation, and every subsequent manualized visit; and HDRS was collected at treatment initiation, at the second baseline visit following the waitlist period for those in the waitlist group, and at every subsequent manualized visit. Clinical assessment raters (*n* = 5) were blind to treatment condition and took part in inter-rater reliability training. The study PI was also blind to treatment condition.

Five sessions of CBT for depression augmented with GDA computer training were then administered over eight weeks starting either within the first week (early) or after a three- to four-week delay (waitlist). Core CBT techniques employed included cognitive restructuring through the use of thought change records; behavioral activation; behavioral probes to examine negative automatic predictions; activity scheduling to increase pleasure and productivity in daily activities; accomplishing challenging tasks by implementing a step-by-step plan; and work to identify and modify more deeply held patterns of negative thinking about oneself, one’s life, and one’s future (“schemas” and “core beliefs”). Study therapists (MT, EH, and MB) were M.D.- and Ph.D.-level therapists with extensive training and experience conducting CBT, including training at the Beck Institute for Cognitive Behavioral Therapy. A participant’s therapist remained the same throughout the duration of their participation.

**Fig. 1:**
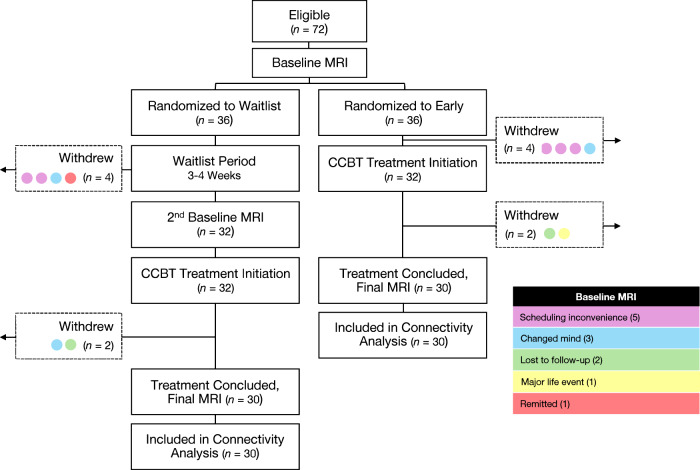
MDD participant flow through the study. Schematic representation of study visits for participants with MDD. MDD major depressive disorder, CCBT computer-augmented cognitive behavioral therapy, MRI magnetic resonance imaging.

Therapy was conducted over eight weeks. The first session with study therapists lasted one hour; subsequent sessions with therapists lasted 30 min. The first virtual session was comprised of two GDA lessons to align with the longer first therapist visit. Therapy then alternated on a weekly basis between manualized CBT with a therapist and a virtual lesson on GDA, culminating in a manualized CBT visit. After the five CBT sessions and nine GDA lessons had been completed, participants had a final debriefing with their assigned therapists. Post-treatment MRI scan and clinical assessments were performed at the conclusion of CBT. Participant flow through the study is depicted in Fig. 1.

### Good days ahead

GDA offered computer-assisted CBT, employing full-screen video and a user-friendly virtual learning environment. The goal of interaction with the program was for individuals to develop and practice CBT skills independently to manage depression and anxiety. Patients learned to identify and modify patterns of dysfunctional thoughts and behaviors through engaging video simulations and self-help exercises. Developed as a tool to support therapists and healthcare professionals in treating emotional disorders, the program’s content is directly rooted in the well-researched CBT pioneered by Aaron T. Beck and his colleagues.

Lisa, the main virtual character navigating common interpersonal and work challenges, is featured in a series of vignettes to illustrate concepts and engage participants. These scenes act as a starting point for various interactive psychoeducational exercises. Users assume three primary roles in the multimedia program: first, an observer, observing Lisa’s transformative journey; second, a learner, receiving instruction on CBT principles; and third, a participant, engaging in CBT skills practice and completing computerized workbook assignments. The program promotes a collaborative clinician–patient relationship, encouraging an empirical approach to problem solving through question-based interactions, mirroring therapist-administered CBT. Organized into nine lessons covering core CBT elements, the program builds on earlier concepts to facilitate a comprehensive understanding of the material in subsequent sections.

### Imaging parameters

Scanning was performed on a Siemens (Erlangen, Germany) 3T Magnetom Prisma Fit with a 64-channel head coil at the University of Pennsylvania. Sequences were rigorously tested as part of the Human Connectome Project [23] and optimized for Siemens Prisma scanners. At each scanning session, 23 min of BOLD resting-state fMRI data were acquired (gradient-echo EPI, 2 mm isotropic resolution, 52° flip angle, 37/800 ms TR/TE) with pepolar field maps. Participants were instructed to keep their eyes on a fixation cross. T1- and T2*-weighted structural images were acquired for functional volume registration (T1w MPRAGE, 0.8 mm isotropic resolution, 8° flip angle, 2400/2.22 ms TR/TE; T2*w SPACE, 0.8 mm isotropic resolution, variable flip angle, 3 200/563 ms TR/TE).

### Image preprocessing

Images were preprocessed with fMRIPrep version 20.0.6 [24] and xcpEngine version 1.1.0 [25] using the preconfigured pipeline fc-36p_despike. Brain tissue was extracted and segmented from anatomical images and normalized to Montreal Neurological Institute (MNI) standard space [26]. BOLD single-band reference images were corrected for head motion using a six-parameter rigid body realignment and then registered to corresponding T1-weighted images using an affine transformation with the BBR cost function [27]. Pepolar field maps were used for susceptibility distortion correction. BOLD timeseries data were resampled into MNI standard space, images were despiked, and confounds were regressed out. Frequencies were bandpass filtered with a 0.01–0.08 Hz passband, voxelwise regional homogeneity was calculated, and spatial smoothing was performed with a 6 mm FWHM SUSAN kernel [28].

The seven-network parcellation from Yeo et al. [29] was used to mask the default mode network (DMN) and frontoparietal network (FPN). A combination of Brodman’s areas 46 and 9 was used to define the left dorsolateral prefrontal cortex (dlPFC). Connectivity was derived between these three masks and the subgenual anterior cingulate cortex (sgACC), bilateral ventral and dorsal anterior insulae, bilateral amygdalae, bilateral hippocampi, bilateral nucleus accumbens, and right dlPFC. To improve SNR of the sgACC, a weighted connectivity seedmap was constructed for Brodman’s area 25 in accordance with methods described by Cash et al. [30]. This seedmap, Brodman’s area 25, and a 10 mm sphere centered around coordinates selected by the Cash et al. method [30] and thresholded through a gray matter mask were used to define the sgACC. The left and right ventral and dorsal anterior insulae were defined using the Kelly et al. [31] *k* = 3 parcellation; left and right insula regions were combined to yield bilateral ventral anterior insulae and bilateral dorsal anterior insulae. The Harvard-Oxford atlas [32] was used for all other regions of interest (ROIs): bilateral amygdalae, bilateral hippocampi, and bilateral nucleus accumbens. Timeseries values within each ROI were averaged across voxels. Connectivity values were computed as *Z*-normalized Pearson correlation coefficients between pairwise timeseries data extracted from all ROIs.

### Cognitive measures

An extensive set of cognitive measures was obtained at baseline and again following CCBT treatment. These data are reported in a separate manuscript.

### Statistical analysis

All analyses were performed using R version 4.3.2 (R Foundation for Statistical Computing, Vienna, Austria). Each participant’s very first study visit, meaning before the waitlist period for those in the waitlist group, was considered the baseline visit for all analyses. Participant demographics were compared between MDD and HC groups and between early and waitlist randomization groups using *t*-tests and Pearson *χ*^*2*^ tests. Baseline connectivity was compared between MDD and HC groups using linear regression models including age, sex, and years of education as covariates. Paired *t*-tests within the waitlist group evaluated MADRS before vs. after the waitlist period. Among participants with MDD, a logistic regression was used to determine whether age, sex, prior depressive episodes (one to two vs. three or more), or randomization group (early vs. waitlist) impacted data completeness due to participant dropout or unusable imaging data. To compare the waitlist and GDA groups on change in depression symptomatology (MADRS and BDI) during treatment, linear mixed-effects models using randomization group and treatment day as main effects and a randomization group x treatment day interaction were constructed; in addition, these models included age, sex, number of prior depressive episodes, and years of education as covariates, together with the corresponding baseline depression score. Similar models using pre- and post-CCBT connectivity scores were used to compare groups on change in connectivity. If significant interactions were not found, analyses were repeated excluding the interaction term. Because the waitlist period did not affect MADRS or BDI scores and randomization group did not interact with treatment day, early and waitlist groups were collapsed into one combined MDD group for connectivity analyses. Pearson partial correlation coefficients were generated to assess the relationship between changes in depression symptomatology (MADRS and BDI) and changes in connectivity, controlling for age, sex, prior depressive episodes, years of education, and randomization group (early vs. waitlist). Post-treatment MDD connectivity was compared to HC connectivity using linear regression models including age, sex, and years of education as covariates. Connectivity changes and between-group differences (MDD vs. HC) were Bonferroni corrected within-ROI (DMN, FPN, and left dlPFC) with a significance cutoff of 0.0167.

MDD participants were classified as responders if they showed a 50% or greater reduction in MADRS. They were classified as remitters if their MADRS scores were 10 or lower. To determine whether responders/remitters and non-responders/non-remitters exhibited different changes in connectivity, connectivity change analyses were repeated first with the addition of treatment response as a covariate, then with the addition of remission status as a covariate; to determine whether treatment response or remission was influenced by any preexisting factors, logistic regressions were performed with age, sex, prior depressive episodes, education, and baseline MADRS score as potential predictors of response or remission status.

## Results

See Table 1 for baseline characteristics and *Z*-normalized connectivity values. Of the 112 enrolled participants, 72 had MDD (36 early group, 36 waitlist group) and 40 were HCs. Eight participants (four early group, four waitlist group) withdrew prior to initiating treatment and four participants (two early group, two waitlist group) withdrew during treatment (see Fig. 1). The remaining participants attended an average of 4.7 out of 5 CBT sessions and reported completing all GDA materials. A logistic regression revealed that data completeness (i.e., having usable imaging data and not dropping out of the study) was not affected by early vs. waitlist group assignment (*z* = −0.12, *p* = 0.91) or any other covariates (see Supplementary information). Controls (ages 19–58 years; 26 female, 14 male) were compared to the initial 72 participants with MDD at baseline before undergoing CCBT treatment (ages 18–60 years; 52 female, 20 male) and to 60 participants with MDD post-treatment (30 early group, 30 waitlist group; ages 18–60 years; 42 female, 18 male). All 60 participants with MDD who completed both baseline and post-treatmtent visits were included in within-group analyses of functional connectivity changes following CCBT; 56 participants with MDD (29 early group, 27 waitlist group; ages 18–60 years; 40 female, 16 male) who completed both baseline and post-treatment visits and had usable MADRS data were included in within-group analyses of MADRS data; 59 participants with MDD (30 early group, 29 waitlist group; ages 18–60 years; 42 female, 17 male) who completed both baseline and post-treatment visits and had usable BDI data were included in within-group analyses of BDI data. Neither age nor sex differed between those with MDD and HCs (age, *t*[90.4] = 0.89, *p* = 0.38; sex, *χ*^*2*^[1] = 0.34, *p* = 0.56). There were no demographic differences between those in the early group and those in the waitlist group. At baseline, there were no differences in connectivity between participants with MDD and HCs.

**Table 1:** Baseline demographic characteristics and connectivity.

	MDD			
Variable	Early (*n* = 36)	Waitlist (*n* = 36)	HC (*n* = 40)	*P*^a^
Sex^b^				0.56
Female	26 (72%)	26 (72%)	26 (65%)	
Male	10 (28%)	10 (28%)	14 (35%)	
Age, years^c^	30 ± 8.7	29 ± 9.8	28 ± 8.0	0.38
Education, years^c,d^	15.5 ± 1.84	15.4 ± 2.46	16.2 ± 1.66	0.04
Prior depressive episodes, *n*^c,e^	3.8 ± 3.88	6.9 ± 9.60	–	–
MADRS score^c,f^	26.7 ± 4.67	26.9 ± 5.23	0.8 ± 1.35	< 0.001
BDI score^c,g^	27.4 ± 8.37	26.8 ± 8.28	1.0 ± 1.32	< 0.001
Connectivity, $r$_*z*_^c^				
L dlPFC				
FPN	0.38 ± 0.19	0.29 ± 0.22	0.29 ± 0.21	0.53
R dlPFC	0.41 ± 0.19	0.38 ± 0.25	0.39 ± 0.21	0.52
sgACC (BA25)	0.12 ± 0.15	0.15 ± 0.11	0.13 ± 0.15	0.41
sgACC (Cash 10 mm sphere)	0.04 ± 0.14	0.07 ± 0.10	0.04 ± 0.14	0.39
sgACC (BA25 seedmap)	0.51 ± 0.21	0.65 ± 0.21	0.54 ± 0.17	0.15
Nucleus accumbens	0.05 ± 0.15	0.06 ± 0.11	0.05 ± 0.11	0.52
Amygdalae	−0.14 ± 0.14	−0.11 ± 0.11	−0.10 ± 0.13	0.18
Hippocampi	−0.08 ± 0.15	−0.05 ± 0.13	−0.05 ± 0.13	0.56
Dorsal anterior insulae	0.02 ± 0.22	−0.05 ± 0.20	0.01 ± 0.17	0.38
Ventral anterior insulae	−0.22 ± 0.18	−0.24 ± 0.17	−0.18 ± 0.15	0.09
DMN				
FPN	0.15 ± 0.19	0.13 ± 0.17	0.12 ± 0.20	0.57
sgACC (BA25)	0.36 ± 0.13	0.39 ± 0.11	0.39 ± 0.15	0.54
sgACC (Cash 10 mm sphere)	0.31 ± 0.13	0.33 ± 0.10	0.32 ± 0.15	0.88
sgACC (BA25 seedmap)	1.09 ± 0.16	1.19 ± 0.18	1.13 ± 0.17	0.64
Nucleus accumbens	0.12 ± 0.13	0.16 ± 0.11	0.15 ± 0.12	0.71
Amygdalae	−0.001 ± 0.13	0.02 ± 0.14	0.04 ± 0.14	0.12
Hippocampi	0.20 ± 0.15	0.20 ± 0.17	0.23 ± 0.15	0.24
Dorsal anterior insulae	−0.28 ± 0.19	−0.31 ± 0.21	−0.32 ± 0.18	0.78
Ventral anterior insulae	−0.41 ± 0.17	−0.40 ± 0.19	−0.39 ± 0.18	0.26
FPN				
DMN	0.15 ± 0.19	0.13 ± 0.17	0.12 ± 0.20	0.57
L dlPFC	0.38 ± 0.19	0.29 ± 0.22	0.29 ± 0.21	0.53
R dlPFC	0.81 ± 0.20	0.82 ± 0.19	0.78 ± 0.20	0.04
sgACC (BA25)	−0.03 ± 0.14	−0.09 ± 0.13	−0.05 ± 0.13	0.81
sgACC (Cash 10 mm sphere)	−0.08 ± 0.13	−0.10 ± 0.11	−0.08 ± 0.12	0.71
sgACC (BA25 seedmap)	0.10 ± 0.19	0.08 ± 0.17	0.09 ± 0.19	> 0.99
Nucleus accumbens	−0.04 ± 0.14	−0.08 ± 0.12	−0.04 ± 0.10	0.13
Amygdalae	−0.25 ± 0.13	−0.30 ± 0.14	−0.29 ± 0.13	0.92
Hippocampi	−0.20 ± 0.15	−0.23 ± 0.16	−0.23 ± 0.15	0.76
Dorsal anterior insulae	0.22 ± 0.16	0.27 ± 0.16	0.25 ± 0.18	0.75
Ventral anterior insulae	−0.10 ± 0.15	−0.07 ± 0.15	−0.08 ± 0.16	0.81

*MDD* major depressive disorder, *HC* healthy control, *MADRS* montgomery-åsberg depression rating scale, *BDI* beck depression inventory–II, *L dlPFC* left dorsolateral prefrontal cortex, *FPN* frontoparietal network, *R dlPFC* right dorsolateral prefrontal cortex, *sgACC* subgenual cingulate cortex, *BA* Brodmann’s area, *DMN* default mode network.

^a^MDD vs. HC. Connectivity pairs were compared using linear models adjusting for age, sex, and years of education. All other characteristics were compared using *t*-tests and *χ*^*2*^ tests.

^b^*n* (%).

^c^Mean ± SD.

^d^*n* = 36 early group, *n* = 35 waitlist group.

^e^*n* = 34 early group, *n* = 33 waitlist group.

^f^*n* = 34 early group, *n* = 35 waitlist group.

^g^*n* = 34 early group, *n* = 36 waitlist group.

### Change in MADRS scores

**Fig. 2:**
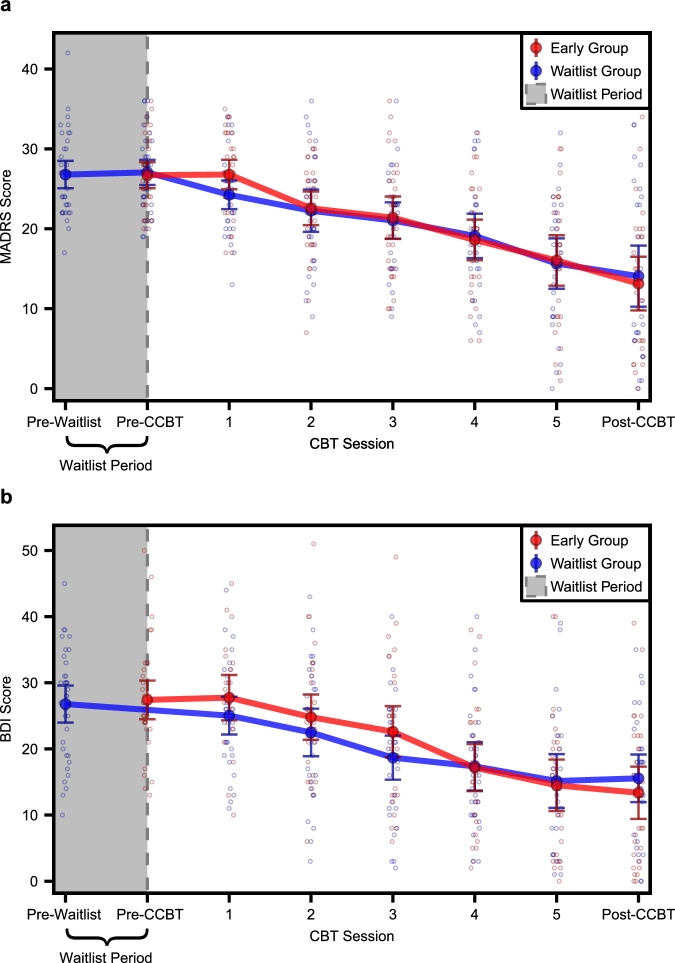
Clinical scores throughout treatment course. Line graphs showing **a** MADRS scores and **b** BDI scores at each study visit for early (red) and waitlist (blue) MDD groups. Solid dots and error bars indicate mean ± 95% CI. MADRS Montgomery-Åsberg Depression Rating Scale, BDI Beck Depression Inventory–II, CBT cognitive behavioral therapy, CCBT computer-augmented cognitive behavioral therapy; pre-waitlist: *n* = 35 waitlist MADRS, *n* = 36 waitlist BDI; pre-CCBT: *n* = 34 early MADRS, *n* = 34 waitlist MADRS, *n* = 34 early BDI; CBT session 1: *n* = 33 early MADRS, *n* = 30 waitlist MADRS, *n* = 24 early BDI, *n* = 32 waitlist BDI; CBT session 2: *n* = 32 early MADRS, *n* = 29 waitlist MADRS, *n* = 31 early BDI, *n* = 31 waitlist BDI; CBT session 3: *n* = 31 early MADRS, *n* = 28 waitlist MADRS, *n* = 30 early BDI, *n* = 31 waitlist BDI; CBT session 4: *n* = 30 early MADRS, *n* = 26 waitlist MADRS, *n* = 30 early BDI, *n* = 28 waitlist BDI; CBT session 5: *n* = 29 early MADRS, *n* = 26 waitlist MADRS, *n* = 30 early BDI, *n* = 27 waitlist BDI; post-CCBT: *n* = 29 early MADRS, *n* = 27 waitlist MADRS, *n* = 30 early BDI, *n* = 29 waitlist BDI.

Within both MDD groups (early and waitlist) combined, the mean ± SD decrease in MADRS from baseline to post-CCBT was 13.4 ± 9.69 points (49.3% average reduction, *n* = 56). Within just the waitlist group, the mean ± SD decrease in MADRS was 0.0 ± 4.37 points (1.7% average reduction, *n* = 33) during the waitlist period and 12.9 ± 9.57 points (48.4% average reduction, *n* = 27) during the eight-week treatment period, with a total decrease from beginning to end of 13.1 ± 9.10 points (49.5% average reduction, *n* = 27). Within just the early group, the mean ± SD decrease in MADRS was 13.8 ± 10.36 points (49.1% average reduction, *n* = 29) from beginning to end (Fig. 2a).

There was no interaction between randomization group (early vs. waitlist) and treatment day on MADRS scores (*F*_1,210.58_ = 0.56, *p* = 0.46). Therefore, post-hoc comparisons were performed using a model that did not include a randomization group x treatment day interaction term. Within this model, there was a main effect of treatment day (*F*_1,211.63_ = 128.76, *p* < 0.001) such that MADRS scores were significantly lower at the end of treatment than at the beginning. In addition, there was a main effect of baseline MADRS scores (*F*_1,46.51_ = 7.10, *p* = 0.01) such that higher baseline MADRS scores were associated with higher MADRS scores throughout treatment. There were no main effects of age (*F*_1,46.57_ = 2.01, *p* = 0.16), sex (*F*_1,47.22_ = 0.07, *p* = 0.07), number of prior depressive episodes (*F*_1,46.59_ = 0.14, *p* = 0.71), years of education (*F*_1,48.20_ = 0.53, *p* = 0.47), or randomization group (*F*_1,46.74_ = 0.27, *p* = 0.61). Within the waitlist group, MADRS scores did not change significantly during the waitlist period (*t*[32] = 0, *p* > 0.99).

### Change in BDI scores

Within both MDD groups (early and waitlist) combined, the mean ± SD decrease in BDI from baseline to post-CCBT was 13.0 ± 9.65 points (47.8% average reduction, *n* = 59). Within just the waitlist group, the mean ± SD decrease in BDI from beginning to end was 11.5 ± 8.60 points (42.1% average reduction, *n* = 29). Within just the early group, the mean ± SD decrease in BDI was 14.6 ± 10.49 points (53.3% average reduction, *n* = 30) from beginning to end (Fig. 2b).

There was no interaction between randomization group (early vs. waitlist) and treatment day on BDI scores (*F*_1,210.82_ = 1.96, *p* = 0.16). Therefore, post-hoc comparisons were performed using a model that did not include a randomization group x treatment day interaction term. Within this model, there was a main effect of treatment day (*F*_1,211.88_ = 146.18, *p* < 0.001) such that BDI scores were significantly lower at the end of the treatment period than at the beginning. In addition, there was a main effect of age (*F*_1,48.31_ = 6.06, *p* = 0.02) such that younger participants had higher BDI scores, as well as a main effect of baseline BDI scores (*F*_1,48.82_ = 48.08, *p* < 0.001) such that higher baseline BDI scores were associated higher BDI scores throughout treatment. There were no main effects of sex (*F*_1,49.33_ = 0.74, *p* = 0.40), number of prior depressive episodes (*F*_1,49.01_ = 0.16, *p* = 0.70), years of education (*F*_1,49.30_ = 0.56, *p* = 0.46), or randomization group (*F*_1,49.00_ = 0.02, *p* = 0.88). HDRS and ISI both decreased significantly following CCBT (see Supplementary information and Supplementary Table 1).

### CCBT connectivity

Because the waitlist period did not affect either MADRS or BDI scores and randomization group did not interact with treatment day, early and waitlist groups were collapsed into one combined MDD group for connectivity analyses.

#### Within-group functional connectivity changes following CCBT

**Fig. 3:**
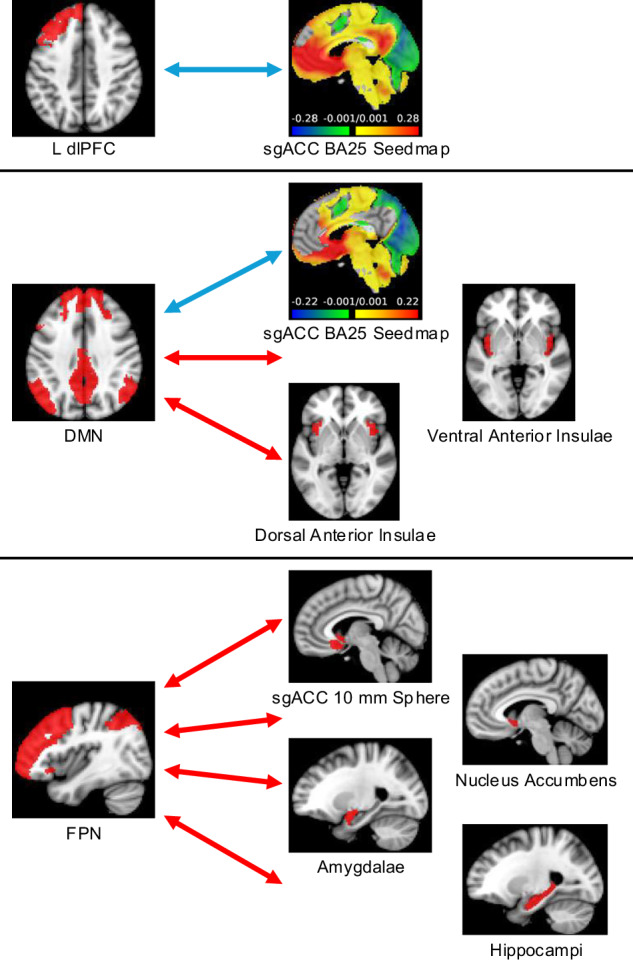
Connectivity changes following CCBT. Regions where connectivity became more positive (red arrows) or more negative (blue arrows) with the L dlPFC (top), DMN (middle), and FPN (bottom) following CCBT. CCBT computer-augmented cognitive behavioral therapy, L dlPFC left dorsolateral prefrontal cortex, sgACC subgenual anterior cingulate cortex, DMN default mode network, FPN frontoparietal network, *n* = 60.

The only connectivity pair with a significant group x time interaction that survived Bonferroni correction was DMN–nucleus accumbens (*F*_1,54_ = 9.14, *p* = 0.004). However, DMN connectivity with nucleus accumbens did not change significantly in either the waitlist group (∆*z* = −0.05, *p* = 0.14) or the early group (∆*z* = 0.07, *p* = 0.06). For all other connectivity pairs, post-hoc comparisons were performed using a model that did not include a group x study visit interaction term, as there was no interaction. After CCBT, the following changes resulting in greater negative correlation occurred in patients with MDD (Fig. 3): the sgACC BA25 seedmap became less positively correlated with the left dlPFC (∆*z* = −0.06, *p* = 0.002) and the sgACC BA25 seedmap became less positively correlated with the DMN (∆*z* = −0.08, *p* = 0.001).

After CCBT, the following positive changes in correlation occurred among those with MDD: the FPN became less negatively correlated with the bilateral nucleus accumbens (∆*z* = 0.06, *p* = 0.01), the bilateral amygdalae (∆*z* = 0.05, *p* = 0.01), the bilateral hippocampi (∆*z* = 0.06, *p* = 0.01), and sgACC 10 mm sphere (∆*z* = 0.06, *p* = 0.01). The DMN became less negatively correlated with the bilateral ventral anterior insulae (∆*z* = 0.09, *p* = 0.001) and the bilateral dorsal anterior insulae (∆*z* = 0.07, *p* = 0.01). No connectivity changes were associated with changes in MADRS scores. However, there was a significant association between change in BDI and change in FPN–sgACC 10 mm sphere connectivity (*partial R*^*2*^ = 0.13, *p* = 0.01). After completing CCBT, there were no connectivity differences between MDD and controls that survived Bonferroni correction (Supplementary Table 2).

#### Effects of response and remission status on connectivity change

Thirty-one out of 56 (55.4%) participants with MDD responded to treatment (16 out of 27 [59.3%] waitlist group, 15 out of 29 [51.7%] early group). Following CCBT, 25 out of 56 (44.6%) participants with MDD remitted (13 out of 27 [48.2%] waitlist group, 12 out of 29 [41.4%] early group). After Bonferroni correction, neither response status nor remission status predicted connectivity change for any connectivity pair. Response status was not predicted by age (*z* = 1.38, *p* = 0.17), sex (*z* = 0.15, *p* = 0.88), prior depressive episodes (*z* = 0.20, *p* = 0.84), randomization group (*z* = 1.15, *p* = 0.25), years of education (*z* = −1.25, *p* = 0.21), or baseline MADRS score (*z* = −0.09, *p* = 0.93); AUC = 0.49. Similarly, remission status was not predicted by age (*z* = 1.38, *p* = 0.17), sex (*z* = 0.51, *p* = 0.61), prior depressive episodes (*z* = −0.03, *p* = 0.98), randomization group (*z* = 1.21, *p* = 0.23), years of education (*z* = −1.63, *p* = 0.10), or baseline MADRS score (*z* = −1.19, *p* = 0.23); AUC = 0.60.

## Discussion

This study achieved a high rate of depression improvement using a five-session shortened schedule of CBT conducted by expert therapists coupled with home computer exercises using the GDA platform. On average, patients improved by 50% from pre-treatment to post-treatment. This result is similar to previous studies using manualized CBT sessions of 12–16 weeks. In both the original proof of concept study of Wright et al. [33] and our large-scale non-inferiority study [34], outpatients with MDD obtained essentially identical benefit from treatment with GDA and 3–5 h of therapist contact as they did with up to 20 h of conventional CBT [11, 34]. In the current study, CCBT intervention provided approximately the improvement expected from previous studies in the literature. Our observed mean improvement of 49% by MADRS criteria and 48% by BDI criteria in the CCBT intervention after eight weeks is a fairly typical outcome in the treatment literature on evidence-based psychotherapies. For example, the outcomes at week 8 in our study are almost identical to the week 8 change in HRDS scores observed in our group’s previous controlled study, which was a noninferiority test vs. conventional CBT [35].

We also searched the literature to benchmark our week 8 results against other groups’ work. In a meta-analysis that looked across several hundred controlled studies of various forms of time-limited, focused psychotherapy—including individual, group, computer-assisted, and internet-based interventions—the average improvement after eight weeks was 41% [36]. Our findings are thus somewhat more favorable than the average result in the literature. Although Cuijpers et al. [36] did not report week 8 outcomes for the subset of CBT studies that examined in-person, individual (conventional) CBT, our Penn colleague Erica Weitz (who is a member of this meta-analysis research group) determined that the average week 8 response rate was 52% in this data set (Weitz, personal communication), which is very comparable to what we observed in the current study. Thus, we have shown that a substantial portion of the therapeutic action of CBT can be shifted from the therapist’s office to the patient’s home or office and, as a result, the cost of treatment could potentially be reduced, while the convenience and accessibility of therapy could be increased.

Further, the repeated practice and mastery of the guided CBT exercises developed for GDA may enhance target engagement at the level of neurocircuitry. The neural mechanisms of CBT have been extensively probed (for reviews, see Messina et al. [37] and Franklin, Carson, and Welch [38]). Many studies have used task-based fMRI to examine correlates of CBT outcome [16, 39]. Previous research also suggests that emotion-based neuroimaging measures might be useful for predicting treatment response. Greater pre-treatment PFC and sgACC activity were reported to predict symptom improvement in CBT across several tasks and internalizing psychopathologies [40–42]. Activity in limbic regions such as the amygdala and anterior insula have also been identified as potential treatment predictors [43]. Some studies have examined longitudinal brain changes accompanying depression improvement [44]. CBT studies in specific diagnostic groups have found that successful treatment decreased limbic neural responsivity to aversive stimuli and enhanced PFC functioning [39, 41, 44].

It is important to note that most of these studies examined task-based markers of emotional reactivity, whereas in studies examining functional connectivity, the opposite direction of outcomes typically occurs. For example, while we previously found that depression treatment was associated with a decrease in amygdala reactivity to negative faces [45], we subsequently identified increased amygdala connectivity to PFC following depression treatment [15], changing from decreased amygdala connectivity at baseline [46]. One interpretation may be that increasing connectivity between central executive network and limbic structures allows for greater cognitive control over subcortical activity. Another interpretation is that our prior finding of increased amygdala connectivity with PFC following depression improvement was due to the transdiagnostic sample that included participants with both post-traumatic stress disorder and MDD. Given the difference in samples, we might not necessarily expect replication.

In the current study, we found that following CCBT, in addition to improvement in depression symptoms, there were changes in cortical–subcortical connectivity. These changes were found across multiple PFC regions, including FPN with nucleus accumbens, bilateral hippocampi, bilateral amygdalae, and sgACC. They were also found in a region within the FPN (dlPFC) with sgACC. The DMN, which is also predominantly prefrontal, demonstrated more positive connectivity with sgACC and ventral and dorsal anterior insulae following CCBT. While there is no specific biomarker for determining brain treatment effects, our results are consistent with the general consensus that changing cortical–subcortical connectivity is associated with depression improvement. Further, we found that change in FPN–sgACC connectivity was correlated with a decrease in depressive symptoms on the BDI, supporting change in connectivity as a correlate of depression improvement. Thus, the mechanism of changes in prefrontal–subcortical connectivity appears to underlie the depression improvement seen with CCBT as well as traditional CBT and seems to be operative even with a shorter treatment duration and lower proportion of time with therapist-guided treatment.

### Study limitations

This study did not have a direct prospective comparison of CCBT with traditional CBT. Therefore, although our study demonstrated similar results to retrospective comparison samples, we cannot say that the treatment efficacy is the same. Additionally, the current study did not find differences in connectivity between HCs and people with MDD, which would have established connectivity as a diagnostic biomarker. However, we do not propose that connectivity functions as a biomarker of illness distinguishing MDD from HCs, but rather as a marker for brain changes induced by therapy as a conduit for clinical improvement—in other words, as an indicator of therapeutic response. This could be considered analogous to using change in heartrate in response to a medication as an indicator of therapeutic response, rather than diagnostic category distinction. In the depression literature, there is a general finding that successful treatment is often accompanied by changes in PFC–subcortical connectivity, which we also found.

In summary, we found that there was no change in depression symptoms during the waitlist period, whereas CCBT was associated with improvement in depression symptoms, both for the group that started on a waitlist prior to CCBT and for the early group that started with CCBT; that CCBT treatment was associated with multiple PFC–subcortical region connectivity changes; and that more positive FPN–sgACC connectivity was associated with a decrease in depression symptoms.

## Supplementary information

Supplemental Material

## Data Availability

The data sets generated during and/or analyzed during the current study are available in the National Institute of Health’s National Data Archive (NDA) repository (ID C2671).
