# Supplementary material for: Neuroimaging changes in major depression with brief computer-assisted cognitive behavioral therapy compared to waitlist
Source: Mol Psychiatry. 2025 Mar 11;30(8):3579–87. doi: 10.1038/s41380-025-02945-x (PMC12240849; doi:10.1038/s41380-025-02945-x)
Supplement: Supplementary file 1 — Supplemental Material [file 41380_2025_2945_MOESM1_ESM.docx]

**Supplementary Information**

Dropout Logistic Regression:

A logistic regression revealed that data completeness (i.e., having usable imaging data and not dropping out of the study) was not affected by early vs. waitlist group assignment (*z* = –0.12, *p* = 0.91), age (*z* = –1.03, *p* = 0.32), sex (*z* = –0.687, *p* = 0.49), or prior depressive episodes (*z* = 0.40, *p* = 0.69); AUC = 0.59. Years of education was not included as a covariate in this analysis because it was recorded after the screening visit, meaning data was insufficiently complete among those who dropped out.

Clinical Measures:

Change in 17-item Hamilton Depression Rating Scale (HDRS) and Insomnia Sleep Index (ISI) scores were compared between GDA and waitlist groups using linear mixed-effects models (LMEs). These LMEs utilized the entire dataset since they were not compared with connectivity values. LMEs to predict HDRS and ISI scores included randomization group and treatment day as main effects and a randomization group x treatment day interaction. They covaried for age, sex, number of prior depressive episodes, and years of education. The ISI LME also covaried for baseline ISI score; HDRS was not administered at baseline. If significant interactions were not found, reported results were derived from models excluding the interaction term. Scores throughout the course of the study are reported in **Supplementary Table 1**.

ISI. There was a significant interaction between randomization group and treatment day on ISI scores (*F*_1,207.14_ = 6.59, *p* = 0.01) such that throughout treatment, ISI scores improved more among those in the early group than they did among those in the waitlist group; therefore, post-hoc comparisons were performed using a model that included a randomization group x treatment day interaction term. Within this model, there were main effects of age (*F_47.23,_* = 6.88, *p* = 0.01), such that younger participants had higher ISI scores, and of baseline ISI scores (*F_1,47.62_* = 59.65, *p* < 0.001), such that higher baseline ISI scores were associated with higher ISI scores throughout treatment. There were no main effects of sex (*F*_1,48.18_ = 3.63, *p* = 0.06), prior depressive episodes (*F_1,48.01_* = 0.07, *p* = 0.80), or years of education (*F_1,48.20_* = 0.07, *p* = 0.79).

HDRS. There was no interaction between randomization group (early vs. waitlist) and treatment day on HDRS scores (*F*_1,212.74_ = 0.13, *p* = 0.72). Therefore, post-hoc comparisons were performed using a model that did not include a randomization group x study visit interaction term. Within this model, there was a main effect of treatment day (*F*_1,213.72_ = 113.72, *p* < 0.001) such that HDRS scores were significantly lower at the end of treatment than at the beginning. There were no main effects of age (*F*_1,48.47_ = 0.53, *p* = 0.47), sex (*F*_1,49.38_ = 3.96, *p* = 0.052), prior depressive episodes (*F_1,48.59_* = 0.43, *p* = 0.51), years of education (*F_1,50.13_* = 0.11, *p* = 0.74), or randomization group (*F*_1,48.75_ = 0.01, *p* = 0.93).

| **Supplementary Table 1: Clinical measures throughout the study. ^1^** | | | | | | |
| --- | --- | --- | --- | --- | --- | --- |
| **Measure** |  | **Early** | |  | **Waitlist** | |
| MADRS ^2^ |  |  |  |  |  |  |
| Pre-waitlist |  | – | |  | 26.9 | ± 5.23 |
| Pre-CCBT |  | 26.7 | ± 4.67 |  | 27.1 | ± 4.78 |
| CBT session 1 |  | 26.8 | ± 5.20 |  | 23.9 | ± 4.84 |
| CBT session 2 |  | 22.6 | ± 5.83 |  | 21.9 | ± 6.95 |
| CBT session 3 |  | 21.4 | ± 7.24 |  | 21.0 | ± 6.31 |
| CBT session 4 |  | 18.6 | ± 6.80 |  | 18.7 | ± 7.12 |
| CBT session 5 |  | 16.0 | ± 8.30 |  | 15.4 | ± 8.37 |
| Post-CCBT |  | 13.1 | ± 8.84 |  | 14.2 | ± 10.29 |
| HDRS ^3^ |  |  |  |  |  |  |
| Pre-waitlist |  | – | |  | – | |
| Pre-CCBT |  | – | |  | 16.5 | ± 3.82 |
| CBT session 1 |  | 15.3 | ± 3.61 |  | 15.4 | ± 3.70 |
| CBT session 2 |  | 14.6 | ± 5.40 |  | 13.0 | ± 4.81 |
| CBT session 3 |  | 12.7 | ± 4.56 |  | 11.9 | ± 4.82 |
| CBT session 4 |  | 10.4 | ± 4.37 |  | 11.1 | ± 4.69 |
| CBT session 5 |  | 9.0 | ± 4.83 |  | 9.2 | ± 5.39 |
| Post-CCBT |  | 7.6 | ± 4.68 |  | 8.9 | ± 6.15 |
| BDI ^4^ |  |  |  |  |  |  |
| Pre-waitlist |  | – | |  | 26.8 | ± 8.28 |
| Pre-CCBT |  | 27.4 | ± 8.37 |  | – | |
| CBT session 1 |  | 27.8 | ± 8.12 |  | 25.0 | ± 7.91 |
| CBT session 2 |  | 24.8 | ± 9.33 |  | 22.5 | ± 9.80 |
| CBT session 3 |  | 22.6 | ± 10.35 |  | 18.7 | ± 9.08 |
| CBT session 4 |  | 17.2 | ± 9.52 |  | 17.4 | ± 9.44 |
| CBT session 5 |  | 14.5 | ± 10.43 |  | 15.1 | ± 10.27 |
| Post-CCBT |  | 13.4 | ± 10.58 |  | 15.6 | ± 9.46 |
| ISI ^5^ |  |  |  |  |  |  |
| Pre-waitlist |  | – | |  | 12.3 | ± 5.62 |
| Pre-CCBT |  | 14.9 | ± 5.07 |  | – | |
| CBT session 1 |  | 14.3 | ± 4.88 |  | 11.7 | ± 5.37 |
| CBT session 2 |  | 12.9 | ± 5.13 |  | 10.7 | ± 5.78 |
| CBT session 3 |  | 11.7 | ± 4.76 |  | 9.2 | ± 5.44 |
| CBT session 4 |  | 9.7 | ± 5.70 |  | 9.4 | ± 5.45 |
| CBT session 5 |  | 7.8 | ± 5.68 |  | 8.5 | ± 5.01 |
| Post-CCBT |  | 8.5 | ± 5.92 |  | 8.9 | ± 5.69 |
| 1. Mean ± SD 2. Pre-waitlist: *n* = 35 waitlist; pre-CCBT: *n* = 34 early, *n* = 34 waitlist; CBT session 1: *n* = 33 early, *n* = 30 waitlist; CBT session 2: *n* = 32 early, *n* = 29 waitlist; CBT session 3: *n* = 31 early, *n* = 28 waitlist; CBT session 4: *n* = 30 early, *n* = 26 waitlist; CBT session 5: *n* = 29 early, n = 26 waitlist; post-CBT: *n* = 29 early, *n* = 27 waitlist 3. Pre-CCBT: *n* = 31 waitlist; CBT session 1: *n* = 32 early, *n* = 29 waitlist; CBT session 2: *n* = 30 early, *n* = 29 waitlist; CBT session 3: *n* = 30 early, *n* = 28 waitlist; CBT session 4: *n* = 29 early, *n* = 26 waitlist; CBT session 5: *n* = 28 early, n = 26 waitlist; post-CBT: *n* = 28 early, *n* = 27 waitlist 4. Pre-waitlist: *n* = 36 waitlist; pre-CCBT: *n* = 34 early; CBT session 1: *n* = 24 early, *n* = 32 waitlist; CBT session 2: *n* = 31 early, *n* = 31 waitlist; CBT session 3: *n* = 30 early, *n* = 31 waitlist; CBT session 4: *n* = 30 early, *n* = 28 waitlist; CBT session 5: *n* = 30 early, n = 27 waitlist; post-CBT: *n* = 30 early, *n* = 29 waitlist 5. Pre-waitlist: *n* = 35 waitlist; pre-CCBT: *n* = 34 early; CBT session 1: *n* = 24 early, *n* = 32 waitlist; CBT session 2: *n* = 31 early, *n* = 31 waitlist; CBT session 3: *n* = 30 early, *n* = 31 waitlist; CBT session 4: *n* = 30 early, *n* = 28 waitlist; CBT session 5: *n* = 30 early, n = 27 waitlist; post-CBT: *n* = 30 early, *n* = 29 waitlist   MADRS Montgomery-Åsberg Depression Rating Scale, CCBT computer–augmented cognitive behavioral therapy, CBT cognitive behavioral therapy, HDRS 17-item Hamilton Depression Rating Scale, BDI Beck Depression Inventory–II, ISI Insomnia Sleep Index. | | | | | | |

| **Supplementary Table 2: MDD post-CCBT connectivity values compared to baseline HC connectivity values.** | | | | | | | | | |
| --- | --- | --- | --- | --- | --- | --- | --- | --- | --- |
| **Connectivity Pair** |  | **Early**  **(*n* = 30)^a^** | |  | **Waitlist**  **(*n* = 30)^b^** | |  | ***P*^b^** | |
| L dlPFC |  |  |  |  |  |  |  |  | |
| FPN |  | 0.40 | ± 0.16 |  | 0.36 | ± 0.20 |  | | 0.10 |
| R dlPFC |  | 0.41 | ± 0.19 |  | 0.45 | ± 0.21 |  | | 0.54 |
| sgACC (BA25) |  | 0.12 | ± 0.13 |  | 0.14 | ± 0.13 |  | > | 0.99 |
| sgACC (Cash 10 mm sphere) |  | 0.06 | ± 0.14 |  | 0.06 | ± 0.14 |  | | 0.60 |
| sgACC (BA25 seedmap) |  | 0.46 | ± 0.17 |  | 0.56 | ± 0.17 |  | | 0.57 |
| Nucleus accumbens |  | 0.09 | ± 0.10 |  | 0.08 | ± 0.12 |  | | 0.25 |
| Amygdalae |  | –0.11 | ± 0.15 |  | –0.13 | ± 0.14 |  | | 0.54 |
| Hippocampi |  | –0.09 | ± 0.11 |  | –0.07 | ± 0.13 |  | | 0.21 |
| Dorsal anterior insulae |  | 0.05 | ± 0.16 |  | –0.01 | ± 0.21 |  | | 0.77 |
| Ventral anterior insulae |  | –0.15 | ± 0.16 |  | –0.22 | ± 0.20 |  | | 0.61 |
| DMN |  |  |  |  |  |  |  | |  |
| FPN |  | 0.13 | ± 0.14 |  | 0.21 | ± 0.14 |  | | 0.29 |
| sgACC (BA25) |  | 0.38 | ± 0.15 |  | 0.34 | ± 0.12 |  | | 0.31 |
| sgACC (Cash 10 mm sphere) |  | 0.34 | ± 0.15 |  | 0.28 | ± 0.12 |  | | 0.73 |
| sgACC (BA25 seedmap) |  | 1.05 | ± 0.21 |  | 1.07 | ± 0.16 |  | | 0.11 |
| Nucleus accumbens |  | 0.18 | ± 0.14 |  | 0.11 | ± 0.13 |  | | 0.88 |
| Amygdalae |  | 0.05 | ± 0.15 |  | 0.01 | ± 0.11 |  | | 0.57 |
| Hippocampi |  | 0.19 | ± 0.11 |  | 0.16 | ± 0.09 |  | | 0.04 |
| Dorsal anterior insulae |  | –0.23 | ± 0.22 |  | –0.23 | ± 0.18 |  | | 0.11 |
| Ventral anterior insulae |  | –0.31 | ± 0.20 |  | –0.32 | ± 0.22 |  | | 0.28 |
| FPN |  |  |  |  |  |  |  | |  |
| DMN |  | 0.13 | ± 0.14 |  | 0.21 | ± 0.14 |  | | 0.29 |
| L dlPFC |  | 0.40 | ± 0.16 |  | 0.36 | ± 0.20 |  | | 0.10 |
| R dlPFC |  | 0.80 | ± 0.18 |  | 0.85 | ± 0.16 |  | | 0.23 |
| sgACC (BA25) |  | –0.03 | ± 0.14 | –0.01 | | ± 0.12 |  | | 0.42 |
| sgACC (Cash 10 mm sphere) |  | –0.04 | ± 0.13 |  | –0.03 | ± 0.14 |  | | 0.20 |
| sgACC (BA25 seedmap) |  | 0.09 | ± 0.13 |  | 0.17 | ± 0.14 |  | | 0.41 |
| Nucleus accumbens |  | –0.03 | ± 0.12 |  | 0.02 | ± 0.12 |  | | 0.60 |
| Amygdalae |  | –0.22 | ± 0.14 |  | –0.23 | ± 0.16 |  | | 0.03 |
| Hippocampi |  | –0.14 | ± 0.17 |  | –0.18 | ± 0.17 |  | | 0.09 |
| Dorsal anterior insulae |  | 0.25 | ± 0.14 |  | 0.26 | ± 0.15 |  | | 0.77 |
| Ventral anterior insulae |  | –0.08 | ± 0.11 |  | –0.10 | ± 0.17 |  | | 0.98 |
| a Mean ± SD *Z*-normalized connectivity values   1. Combined MDD groups compared to HC using linear models adjusting for age, sex, and years of education   MDD major depressive disorder, HC healthy controls, CCBT computer–augmented cognitive behavioral therapy, L dlPFC left dorsolateral prefrontal cortex, FPN frontoparietal network, R dlPFC right dorsolateral prefrontal cortex, sgACC subgenual cingulate cortex, BA Brodmann’s area, DMN default mode network. | | | | | | | | | |
